# A Review of the Association of Blue Food Coloring With Attention Deficit Hyperactivity Disorder Symptoms in Children

**DOI:** 10.7759/cureus.29241

**Published:** 2022-09-16

**Authors:** Rachel M Rambler, Erica Rinehart, Wendy Boehmler, Prerna Gait, Joan Moore, Melissa Schlenker, Rahul Kashyap

**Affiliations:** 1 Research, WellSpan Health, York, USA; 2 Biological Sciences, York College of Pennsylvania, York, USA; 3 Medical School, St. George’s University School of Medicine, True Blue, GRD; 4 Research, Global Clinical Scholars Research Training, Harvard Medical School, Boston, USA

**Keywords:** artificial food coloring, attention deficit hyperactivity disorder, blue no.2, blue no.1, blue food coloring

## Abstract

Attention Deficit Hyperactivity Disorder, also known as ADHD, is a neurodevelopmental disorder diagnosed in children. The exact cause of ADHD is not known, but, along with genetic factors, it is possible that environmental factors including toxins and diet may affect symptom severity. Of these dietary components, artificial food coloring (AFC), while approved by the Food and Drug Administration (FDA), has been suspected to be associated with ADHD symptoms. Of the nine FDA-certified food colors, two are used for artificial blue coloring: Blue No. 1 and Blue No. 2. There is limited literature describing the possible role of blue AFC in causing symptoms of ADHD in children. This paper provides a review of the literature surrounding artificial food coloring and its ability to affect the neurodevelopment of children in a way that could increase the behavioral indicators of ADHD. To do this, search criteria were established using a combination of MeSH terms about blue AFCs and ADHD and were entered into PubMed, along with limits on article types and publication dates from January 2000 to June 2022. There was a total of 20 articles that met this search criterion. These articles were reviewed by authors, and the ones not relevant to the topic were excluded. In total, four studies were chosen to be included in this article. After reviewing the literature, it was found that restriction diets, specifically those excluding AFCs, may affect symptom severity. The source of these changes is not known, but possible mechanisms include AFCs causing nutritional deficiencies and allergic reactions or altering neurotransmitter levels. More research is necessary to describe the neurotoxicity of artificial blue dyes in humans.

## Introduction and background

Attention Deficit Hyperactivity Disorder, also known as ADHD, is a neurodevelopmental disorder that is one of the most common disorders of its type in the United States, with a worldwide prevalence of 3.4% [1]. Since those with the condition display a variety of different symptoms with different levels of severity, a diagnosis of ADHD is reached by considering a combination of several traits, such as impulsivity, short attention span, and hyperactivity [2]. While the exact cause of ADHD is not known, it is thought to originate from a combination of genetic factors, as well as environmental factors that include toxins and dietary components that have not yet been proven to have a causal relation [3]. Of these dietary components, artificial food coloring (AFC), while approved by the Food and Drug Administration (FDA), has been suspected to be associated with ADHD symptoms since the 1970s [4]. Since then, there have been several studies to show that some children with ADHD experience a decrease in symptoms when not consuming any AFCs [5].

Of the nine FDA-certified food colors, two are used for artificial blue coloring: Blue No. 1, also known as Brilliant Blue FCF, and Blue No. 2, also known as Indigotine. These artificial blue coloring agents are used in beverages, ice cream, candy, and baked goods. Blue No. 1, along with its structural analogs such as Brilliant Blue G, is the only AFC that the blood-brain barrier is known to be permeable to[6]. Despite this unique characteristic, there is limited literature describing the possible role of blue AFC in causing symptoms of ADHD in children. While there are several studies that examine the effect of a combination of AFCs on ADHD symptoms in children, there is much less research done on just blue artificial food coloring in children. This paper provides a review of the literature surrounding artificial blue food coloring and its ability to affect the neurodevelopment of children in a way that could increase the behavioral indicators of ADHD.

## Review

**Figure 1:**
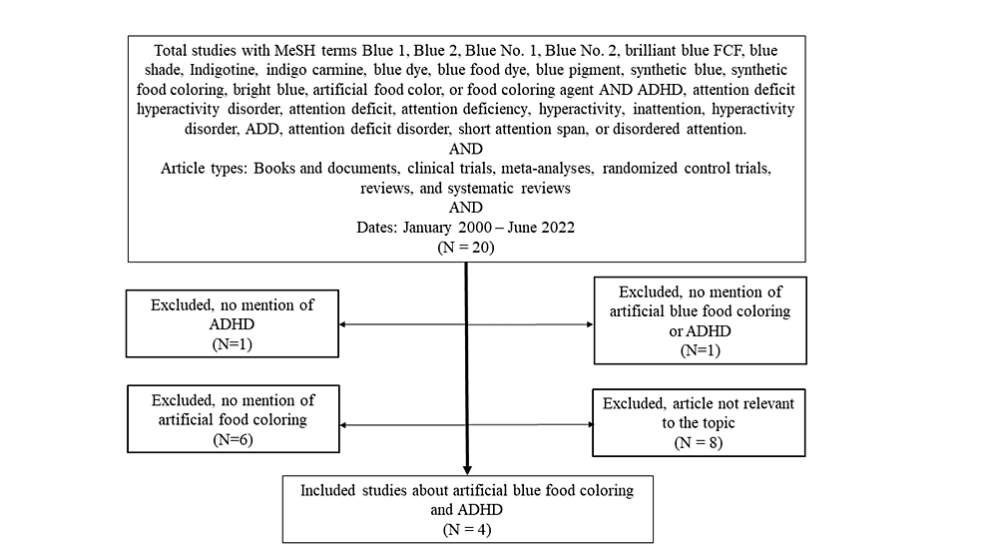
Flow diagram of both included and excluded articles used in the study ADHD: Attention Deficit Hyperactivity Disorder; MeSH: Medical Subject Heading; ADD: Attention Deficit Disorder

For this review on artificial blue food coloring and ADHD symptoms in children, we established search criteria using a combination of MeSH terms. Those terms consisted of ‘Blue 1’, ‘Blue 2’, ‘Blue No. 1’, ‘Blue No. 2’, ‘brilliant blue FCF’, ‘blue shade’, ‘Indigotine’, ‘indigo carmine’, ‘blue dye’, ‘blue food dye’, ‘blue pigment’, ‘synthetic blue’, ‘synthetic food coloring’, ‘bright blue’, ‘artificial food color’, or ‘food coloring agent’ and the terms ‘ADHD’, ‘attention deficit hyperactivity disorder’, ‘attention deficit’, ‘attention deficiency’, ‘hyperactivity’, ‘inattention’, ‘hyperactivity disorder’, ‘ADD’, ‘attention deficit disorder’, ‘short attention span’, or ‘disordered attention’ (Figure 1). These terms were entered into PubMed along with the following limits: article type including ‘books’ and ‘documents’, ‘clinical trials’, ‘meta-analyses’, ‘randomized control trials’, ‘reviews’, and ‘systematic reviews’ published from January 2000 to June 2022.

These articles were screened by authors R.R. and E.R. and evaluated for pertinence and inclusion. Any disagreement between initial reviewers was resolved by mutual consensus and consultation with senior reviewer R.K. There were a total of 20 articles that met this search criterion. These articles were reviewed, and some were excluded for the following reasons: not relevant to the topic (n=8), no mention of artificial food coloring (n=6), no mention of ADHD (n=1), or no mention of ADHD or artificial blue food coloring (n=1). This process is described in Figure 1. In total, four studies were chosen to be included in this article’s discussion, and the details about the studies are described in Table 1.

**Table 1:** Summary of included studies in literature review ADHD: Attention Deficit Hyperactivity Disorder

Author	Title	Study Type	Year	Studied AFC = 1, Blue AFC = 2	Studied ADHD (No = 0, Yes = 1)
Pelsser et al.	Diet and ADHD, Reviewing the Evidence: A Systematic Review of Meta-Analyses of Double-Blind Placebo-Controlled Trials Evaluating the Efficacy of Diet Interventions on the Behavior of Children with ADHD	Systematic Review of Meta-Analyses	2017	1	1
Nigg et al.	Meta-Analysis of Attention-Deficit/Hyperactivity Disorder or Attention-Deficit/Hyperactivity Disorder Symptoms, Restriction Diet, and Synthetic Food Color Additives:	Meta-analysis	2015	1	1
Bateman et al.	The effects of a double-blind, placebo-controlled, artificial food colourings and benzoate preservative challenge on hyperactivity in a general population sample of preschool children	Randomized control trial	2004	1	1
Miller et al.	Diet and ADHD, Reviewing the Evidence: A Systematic Review of Meta-Analyses of Double-Blind Placebo-Controlled Trials Evaluating the Efficacy of Diet Interventions on the Behavior of Children with ADHD	Systematic Review	2022	1, 2	1

Diets and ADHD

While pharmacological intervention is common for children with ADHD, there is an increasing number of parents who would prefer a treatment option that does not require pharmaceuticals, if possible [7]. One of these other options is changing components of a child’s diet to see if there is a change in behavioral symptoms. Dietary elements that have been implicated in the symptom onset of ADHD include deficiencies in iron, copper, and manganese [8] fatty acids, and artificial food coloring. The growing interest in the role of diet in ADHD management has led to an increase in restriction diet studies, a type of study in which one component is removed from the participant’s diet and any resulting behavior change is noted. Two out of four studies that were the result of the aforementioned article search examined restriction diets and their effects on ADHD symptoms in children.

The article by Nigg et al. [9] looked into studies that utilized restriction diets that limited the amounts of synthetic color additives, flavors, and salicylates in the diets of children with ADHD. Their analysis concluded that these restriction diets did reduce the hyperactive symptoms in approximately 33% of the children with ADHD that were involved. Pelsser et al. [10] examined meta-analyses of double-blind placebo-controlled studies on three types of altered diets: elimination of artificial food color (AFC), the addition of polyunsaturated fatty acids (PUFAs), and the few-foods diet (FFD) that limits a person’s diet to more uncommon foods with low potential to be allergenic. While the study found that the FFD was shown to lessen inattention and hyperactivity in children with ADHD, the effect of AFCs on ADHD symptoms was inconclusive and further research on the topic was suggested.

AFCs must be approved by the FDA before being used as a food additive in the United States. Data from the FDA shows that consumption of AFCs has been steadily increasing in the United States as more foods are being made to look brighter and more appetizing to consumers [11]. Among studies that test the effects of general restriction diets on ADHD symptoms, some test for differences in symptoms when only AFCs are excluded from diets of those with ADHD. Two out of four articles that resulted in the search methods used in this review contained studies of this sort. Nigg et al. [9], in a study that reviewed AFC-exclusion diets and their effect on ADHD, reported that approximately 8% of children with ADHD could experience an exacerbation of hyperactivity and inattention symptoms when AFCs are included in their diets. This was based on the observations from parents, who rated their child’s ADHD symptoms, such as hyperactivity, after food color additives were removed from the child’s diet.

A double-blind placebo-controlled study done by Bateman et al. [12] specifically observed three-year-old children who showed symptoms of ADHD such as hyperactivity. The children, after having a baseline assessment, maintained an AFC exclusion diet, followed by a period of time with either an AFC and sodium benzoate-supplemented drink added to their diet or a placebo drink. The children were then rated on hyperactivity level by parents and a tester blind to their dietary status. The study found that when the participants had AFCs and sodium benzoate eliminated from their diet, there were significant reductions in hyperactive behavior. This corresponded with increased hyperactive behavior in children taking the AFC-benzoate supplementation drink compared to those with the placebo drink. This finding suggests that a combination of AFCs and sodium benzoate, which is also able to cross the blood-brain barrier [13], did contribute to hyperactive symptoms in three-year-old children. 

Effects of blue artificial food coloring

In foods that are artificially colored blue, FDA-approved synthetic additives Blue No. 1 and Blue No. 2 can be found. Blue No. 1, also called Brilliant Blue, Brilliant Blue FCF (for coloring food), and FD&C Blue No. 1, is used to add a bright, neon blue color mainly to food but additionally to drugs and cosmetics. Blue No. 2, also called Indigo Carmine, Indigotine, and FD&C Blue No. 2, is derived from indigo and is also used in food, drugs, and cosmetics to give a darker blue-indigo color. The accepted daily intake (ADI) for Blue No. 1, a value established by the FDA in 1969 that indicates the amount of an AFC that is safe to consume throughout the day, is 12.0 mg/kg/day, and is higher than the ADI for Blue No. 2, which is 2.5 mg/kg/day [11].

The toxicity of artificial blue food coloring has been studied in mice and rats in laboratory settings as questions about the safety of AFCs continue to rise. A study was done in rats to test for the carcinogenicity of Blue No. 2, but the results concluded that while there was an increase in gliomas in the rats given a high dose of Blue No. 2, these changes did not meet the requirements to be considered neurocarcinogenic since the trends were consistent with historical controls [14]. In regard to neurotoxicity, a study done with neuroblastoma cells treated with Blue No. 1, along with food additive glutamic acid, showed a statistically significant inhibition of neurite growth when Blue No. 1 was added to the cells [15]. As for development, a study in mice that examined neurobehavioral development between mice given different doses of Blue No. 1 found that the increase in the time it took for mice given a high dose of Blue No. 1 to perform coordinated movement tasks was statistically significant [16]. The study also found a decrease in exploratory behavior and an increase in spontaneous and hyperactive behavior in female mice given high doses of Blue No. 1.

The effects of artificial blue dyes Blue No. 1 and Blue No. 2 have also been studied in children, along with other AFCs. A study done by Miller et al. [17], which was a study included in the results for the article search for this paper, reviewed 27 clinical trials that assessed hyperactivity and/or inattention in children given known quantities of synthetic food dyes, including blue dyes. The study found that 52% of the clinical trials found an association between intake of artificial food coloring and behavioral responses such as hyperactivity and inattention that were statistically significant.

Mechanism of action/pathophysiology

Since ADHD does not have a known etiology, it is not immediately clear how blue AFC could cause increased hyperactivity or inattentiveness in children. One possible explanation is that artificial food coloring could cause nutritional deficiencies that could lead to impaired neuronal development. One study showed that adding yellow AFC to the diets of male children both with and without ADHD resulted in a decrease in zinc levels in serum and blood, along with worsening behavioral symptoms in children with ADHD [18]. Zinc deficiency may lead to worsening ADHD symptoms due to decreased neuroplasticity, which is crucial for neurodevelopment, and because of a possible buildup of heavy metals [19]. For example, zinc deficiency has been linked to mercury toxicity because mercury can build up in the body, such as from consuming foods containing high levels of mercury. When this happens, a person with normal zinc levels will produce metallothionein. This compound helps to metabolize and eliminate the mercury that builds up in the body. Without sufficient zinc to fuel this process, mercury can accumulate and cross the blood-brain barrier. This effect may be further increased by zinc deficiency, which is associated with a loss in blood-brain barrier integrity in zinc-deficient mice [20]. Since studies have shown that heavy metals like mercury may cause susceptibility to ADHD in children due to its effect on neurodevelopment [21], the zinc deficiency that AFCs can cause could be a factor in ADHD symptom exacerbation. This is further supported by studies that have shown that treating patients with ADHD with zinc sulfate decreased hyperactive and impulsive symptoms in those patients [22]. It is again important to note that Blue No. 1 and its analogs can cross the blood-brain barriers even in adults with mature barriers [6], potentially making the brain even more susceptible to metal toxicity.

The ability of AFCs to alter levels of trace elements in the body like zinc and manganese may also affect people with ADHD because of these elements’ role in the creation and breakdown of neurotransmitters in the central nervous system (CNS). The neurotransmitter dopamine has been thought to be associated with ADHD symptoms because the chemical is involved in the executive functions of the brain that people with ADHD struggle with [23]. Medication options include drugs such as the psychostimulant methylphenidate, which promotes the brain’s release of dopamine and blocks dopamine transporters [24]. Since the trace element manganese impedes the ability of enzymes to regulate dopamine levels [8], consuming AFCs that could increase the amount of manganese in one’s body could lead to decreased dopamine levels and worsened ADHD symptoms.

Since the link between artificial food coloring and ADHD was made in the 1970s [4], there has been a hypothesis that allergies and hypersensitivity to AFCs could be responsible for the behavioral changes seen in some children who consume the dyes. Histamines, which are chemicals part of the immune system that are released in the presence of an allergen that causes sneezing, itching, and other symptoms, are released in some children after consuming AFCs [25]. The study by Bateman et al. [12], which was previously mentioned as a result of the article search done for this study, tested for an association between atopy, which is an amplified immune response mediated by IgE antibodies, and increased hyperactivity in response to consuming AFCs. Their results concluded that the effect of AFCs was not dependent on IgE histamine release, and most likely would fit in with the non-IgE dependent histamine release mechanism suggested by other studies on food additives and immune response [26]. One study in which three-to-four- and eight-to-nine-year-old children were given AFCs, although not containing blue food dyes, also had genotyping done in the participants to look for single nucleotide polymorphisms (SNPs) in genes that have been indicated in symptoms of ADHD because of their effect on histamine and dopamine [27]. The study found that SNPs in the *HNMT* gene, which codes for a protein involved in the metabolism of histamine, were linked to hyperactive behavior and sensitivity to AFCs in the participants. This shows that the body’s response to histamine could play a role in the extent to which AFCs may affect a child with ADHD, though further testing with only blue AFCs would need to be done to determine its specific effect.

A limitation of studies on blue artificial food coloring is that some food and beverages that contain the dye are high in refined sugar as well, which may also play a role in ADHD symptom exacerbation. A meta-analysis that assessed how ADHD was affected by dietary patterns found that diets high in refined sugar increased the risk of ADHD or hyperactivity [28]. Without controlling refined sugar levels, it is difficult to determine whether the worsening of ADHD symptoms seen after consuming foods like ice cream, candy, and soda that contain blue AFCs is not at least partially due to the sugar content.

Implications for treatment and future directions

For treating children with ADHD, pharmacological intervention is widely used. In many countries, including the United States, the use of psychostimulants to treat ADHD is recommended, sometimes before behavioral management is considered [29]. Stimulant medications include methylphenidate and amphetamine, along with norepinephrine and dopamine reuptake inhibitors, which both come in different dose ranges and delivery types [30]. Non-stimulant medications that are also used include atomoxetine, a selective norepinephrine reuptake inhibitor, and clonidine, an α2-adrenergic agonist. However, medication adherence has been found to decline in children with ADHD with prolonged use [31], leaving the question as to how effective this type of treatment is for children with the disorder.

The studies that were reviewed for this article support the suggestion that dietary intervention, particularly the elimination of AFCs, should be considered for children with ADHD. The study by Nigg et al. [9] concluded that 8% of the children with ADHD in the studies they reviewed were affected by AFCs and that 33% of the children with ADHD could benefit from dietary intervention including other synthetic food additives. Bateman et al. [12] had similar findings, suggesting that eliminating AFCs from children’s diets could cause a significant improvement in the hyperactive behavior associated with ADHD. However, that study also found that initial levels of hyperactivity were not related to the degree of behavioral change noticed after removing AFCs from a child’s diet, suggesting that many children, not just those diagnosed with ADHD, may benefit from such a dietary change.

## Conclusions

The studies that were reviewed in this article show that diet, especially consumption of artificial food coloring, produces statistically significant increases in ADHD symptoms in children. The mechanism for how AFCs cause such neurological and behavioral changes is not definitively known, but there are a few possible explanations. These include some well-studied hypotheses for the mechanism of ADHD that AFCs could contribute to, such as the dopamine hypothesis, but some less-studied causes like nutritional deficiencies and histamine release may also be possible. While several studies examine the effect of a combination of AFCs on ADHD symptoms in children, there is very little research done on just Blue No. 1 or Blue No. 2 in children. This is illustrated by the low number of studies reviewed in the article after the PubMed search on blue AFC and ADHD, limiting the strength of the conclusions that can be made from the results. In mice and rats, Blue No. 1 specifically has been found to affect neurodevelopment and hyperactive behavior, while Blue No. 2 has not been shown to have any definitive toxicity. There is a need for more research to determine how these individual compounds affect humans, especially because of Blue No. 1’s ability to cross the blood-brain barrier. Understanding how artificial blue food dyes affect the brain and behavior of humans through increased research on the topic could provide alternative treatment options for children with ADHD.
